# Deep learning signature to predict postoperative anxiety in patients receiving lung cancer surgery

**DOI:** 10.3389/fsurg.2025.1573370

**Published:** 2025-03-24

**Authors:** Qingqing Ji, Guohua Zhou, Xiangxiang Sun

**Affiliations:** ^1^ Shanghai University of Engineering Science, Shanghai, China; ^2^ Department of Anesthesiology, Ningbo First Hospital, Ningbo, Zhejiang, China; ^3^ Department of Thoracic Surgery, The First Affiliated Hospital of USTC, Division of Life Sciences and Medicine, University of Science and Technology of China, Hefei, Anhui, China

**Keywords:** deep learning, biomarker, postoperative anxiety, lung cancer, surgical resection

## Abstract

This study aims on establishing and validate a deep learning signature based on magnetic resonance imaging (MRI) to predict postoperative anxiety in patients receiving lung cancer surgery. In the current study, 202 patients receiving lung cancer surgery were included. Preoperative MRI-T1WI images were collected to train the deep learning signature utilized the ResNet-152 algorithm. The relationships between clinical variables and postoperative anxiety were explored via Logistic regression and the predictive performances of the developed deep learning signature were evaluated via receiver operating characteristic analysis. Larger tumor size [odds ratio (OR), 2.044; 95% confidence interval (CI), 1.736–3.276; *p* = 0.002] and occurrence of lymph node metastasis (OR, 2.078; 95% CI, 1.023–3.221; *p* = 0.043) were revealed as independent predictors for postoperative anxiety. With the increase of deep learning scores, more patients experiencing postoperative anxiety were identified. Moreover, our deep learning signature yielded areas under the curve of 0.865 (95% CI, 0.800–0.930) and 0.822 (95% CI, 0.695–0.950) to predict postoperative anxiety. Therefore, our deep learning signature could help identify lung cancer patients with high risks of postoperative anxiety.

## Introduction

Globally, lung cancer ranks among the most common solid tumors and is a leading cause of cancer mortality. Its development is strongly associated with various risk factors, such as tobacco use, chronic obstructive pulmonary disease, and genetic predisposition (1–3). While advancements in radiotherapy, targeted treatments, and immunotherapy have partially enhanced clinical outcomes for lung cancer patients (4–6), mental health challenges remain a significant concern, adversely impacting their quality of life and survival rates (7, 8). Consequently, investigating the psychological issues faced by these patients is essential for optimizing their care.

Anxiety is a widespread mental health condition that negatively influence lung cancer patient outcomes. These disorders affect between 20.9% and 57.1% of individuals with lung cancer (9–11) and have been identified as indicators of poorer survival (12). Research indicates that post-surgical lung cancer patients experience anxiety influenced by factors like gender, marital status, complications, and aggressive tumor characteristics, all of which correlate with worse prognoses. Hence, precise recognition of patients who have the risk to experience anxiety after surgery is crucial for personalized management of lung cancer.

Deep learning-based radiomics, which extracts detailed features from medical images, offers a promising tool for tumor diagnosis, prognosis, and treatment planning (13–16). We hypothesized that the deep learning technique could capture features associated with postoperative anxiety from medical imaging, and further quantify the risks of postoperative anxiety to optimize the management of lung cancer. Therefore, this study purposes to develop and validate an imaging signature to predict anxiety based on the deep learning algorithm in patients after lung cancer surgery.

## Materials and methods

### Study population and data collection

**Figure 1:**
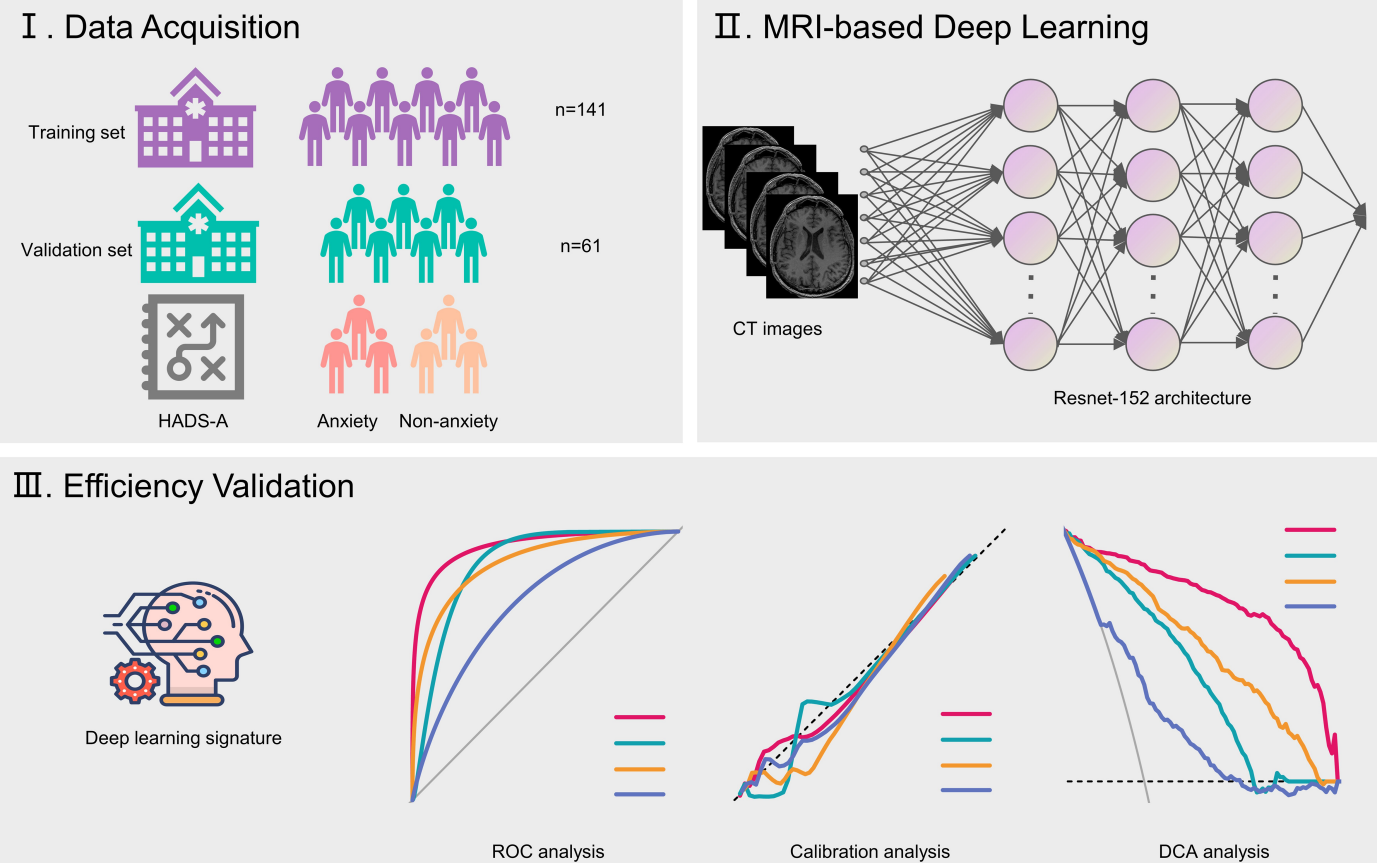
Flow chart illustrating study design. HADS-A, Hospital Anxiety and Depression Scale-Anxiety.

The study obtained approval from the institutional review boards and ethics committees at Ningbox First Hospital. Patients undergoing lung cancer surgery between January 2024 and December 2024 were consecutively recruited. Clinicopathological information was extracted from electronic medical records. Anxiety was evaluated 3 days post-surgery using the Hospital Anxiety and Depression Scale-Anxiety (HADS-A), where scores range from 0 to 21, and a score ≥ 8 signifies anxiety (17). Preoperative brain magnetic resonance imaging (MRI) scans, conducted within 1 week before surgery, were obtained from the picture archiving and communication system. The study's design is depicted in Figure 1.

### Deep learning procedures

All MRI-T1WI scans were download as DCM format. The personal information of patients in MRI images including name, medical number and hospital name were eliminated and images were transformed into NIfTI format by using an in-house software. In order to analyze images in an isotropic voxel size, all images were resampled to the voxel size of 1 mm × 1 mm × 1 mm. Brain MRI NII format images were segmented using the MedSAM2 algorithm (18) and the segmented data were reviewed by a senior radiologist to ensure the accuracy of segmentation. Each segmented MRI was annotated by a specific anxiety label.

**Figure 2:**
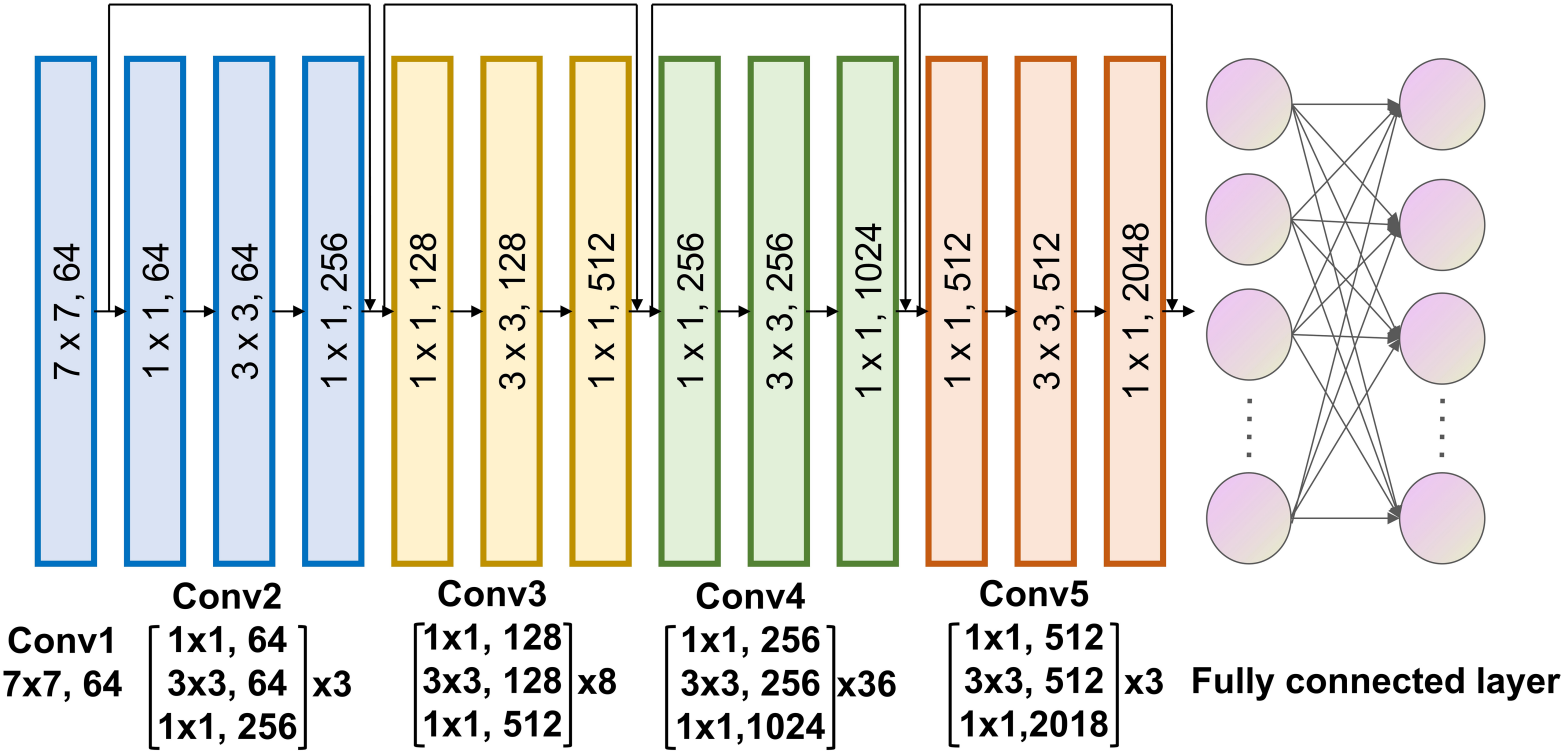
Architecture of the deep learning algorithm.

Our deep learning model was developed using ResNet-152 (Figure 2). The input consisted of segmented MRI scan data, and the algorithm generated probability outputs for various categories. The entire cohort was divided in to a training and validation set at the ratio of 7:3. The Softmax cross-entropy loss between predictions and ground truth labels was minimized using a momentum optimizer, with a batch size of 64 and an initial learning rate of 0.01. The learning rate was reduced every 300 iterations using an exponential decay rate of 0.99. Data augmentation techniques included random rotations (0°, 90°, 180°, 270°) along the *Z*-axis and random flips across the *X*, *Y*, and *Z* axes. L2 regularization was applied to mitigate overfitting. Training concluded after 3,000 iterations, with the model exhibiting the lowest loss being selected. This AI model was specifically designed to classify anxiety vs. non-anxiety cases. Training was conducted on a system equipped with an NVIDIA GTX 4070 GPU (NVIDIA, Santa Clara, CA) and leveraged the TensorFlow framework (Google, Mountain View, CA). Python 3.6.4 was used for all programming tasks.

### Statistical analysis

Baseline data were summarized as frequencies (percentages) for categorical variables and means ± standard deviations for numerical variables. Comparisons were made using the Chi-square test for categorical data and the *t*-test for numerical data. Model performance was evaluated using receiver operating characteristic (ROC) curves, with areas under the curve (AUCs) calculated for further analysis. Univariate and multivariate logistic regression analyses were performed to identify clinical predictors for anxiety, with a clinical model constructed using backward elimination. The performance metrics including sensitivity, specificity, positive predictive value (PPV), negative predictive value (NPV), and accuracy were determined by the maximum Youden index in the training set. All statistical analyses were conducted using R (version 4.3.1) and Python (version 3.6.4), with a *p*-value <0.05 considered statistically significant.

## Results

### Clinicopathologic characteristics

Table 1 summarizes the clinicopathological characteristics of the study population. The cohort comprised 94 males (46.5%) and 108 females (53.5%), with an average age of 59.4 years. A history of smoking was reported in 30 patients (14.8%). Adenocarcinoma was the most common histological type, representing 81.2% (*n* = 164) of cases. Tumors were predominantly located on the right side (57.9%, *n* = 117), with an average size of 1.8 cm. Lymph node metastasis was detected in 22 patients (10.9%). Based on the HADS-A scale, 80 individuals (39.6%) were categorized as having anxiety. Subgroup analysis comparing the training (*n* = 141) and the validation groups (*n* = 61) revealed no statistically significant differences between the two groups.

**Table 1:** Baseline characteristics of included patients.

Characteristics	Entire cohort (*n* = 202)	Training (*n* = 141)	Validation (*n* = 61)	*p* value
Age (years), mean ± SD	59.1 ± 11.0	58.9 ± 11.8	59.6 ± 9.2	0.696
Sex, *n* (%)				0.298
Male	94 (46.5)	69 (48.9)	25 (41.0)	
Female	108 (53.5)	72 (51.1)	36 (59.0)	
Smoking, *n* (%)				1.000
Ever	30 (14.8)	21 (14.9)	9 (14.7)	
Never	172 (85.2)	120 (85.1)	52 (85.3)	
Histology, *n* (%)				0.395
Squamous cell carcinoma	24 (11.6)	19 (13.5)	5 (8.2)	
Adenocarcinoma	164 (81.2)	111 (78.7)	53 (86.9)	
Others	14 (6.9)	11 (7.8)	3 (4.9)	
Location, *n* (%)				0.147
Left	85 (42.1)	61 (45.4)	21 (34.4)	
Right	117 (57.9)	77 (54.6)	40 (65.6)	
Tumor size, mean ± SD	1.8 ± 0.7	1.8 ± 0.7	1.9 ± 0.7	0.293
Lymph node metastasis, *n* (%)				0.867
No	180 (89.1)	125 (88.7)	55 (90.2)	
Yes	22 (10.9)	16 (11.3)	6 (9.8)	
Anxiety assessed by HADS-A, *n* (%)				0.960
Yes	80 (39.6)	56 (39.7)	24 (39.3)	
No	122 (60.4)	85 (60.3)	37 (60.7)	
Surgical approach, *n* (%)				0.865
Thoracoscopic	196 (97.0)	137 (97.2)	59 (96.7)	
Thoracotomy	6 (3.0)	4 (2.8)	2 (3.3)	

HADS-A, Hospital Anxiety and Depression Scale-Anxiety; SD, standard deviation.

### Variables associated with postoperative anxiety

In the univariable analyses (Table 2), larger tumor size [odds ratio (OR), 2.334; 95% confidence interval (CI), 1.546–3.479; *p* = 0.003] and occurrence of lymph node metastasis (OR, 1.999; 95% CI, 1.133–3.528; *p* = 0.017) were significantly associated with postoperative anxiety. Similarly, in the multivariable analyses, larger tumor size (OR, 2.044; 95% CI, 1.736–3.276; *p* = 0.002) and occurrence of lymph node metastasis (OR, 2.078; 95% CI, 1.023–3.221; *p* = 0.043) independently predicted postoperative anxiety.

**Table 2:** Logistic analyses for postoperative anxiety.

Variables	Anxiety			
	Univariable		Multivariable	
	OR (95% CI)	*p* value	OR (95% CI)	*p* value
Age	0.994 (0.969–1.020)	0.625		
Sex (Male)	1.108 (0.629–1.951)	0.723		
Smoking history (Ever)	0.813 (0.367–1.803)	0.611		
Histology (Adenocarcinoma)	0.539 (0.141–2.033)	0.359		
Location (Left)	1.058 (0.597–1.874)	0.847		
Tumor size	2.334 (1.546–3.479)	0.003	2.044 (1.734–3.276)	0.002
Lymph node metastasis (Yes)	1.999 (1.133–3.528)	0.017	2.078 (1.023–3.221)	0.043
Surgical approach (Thoracoscopic)	0.943 (0.903–1.102)	0.878		

OR, odds ratio; CI, confidence interval.

### Predictive performance of deep learning signature

**Figure 3:**
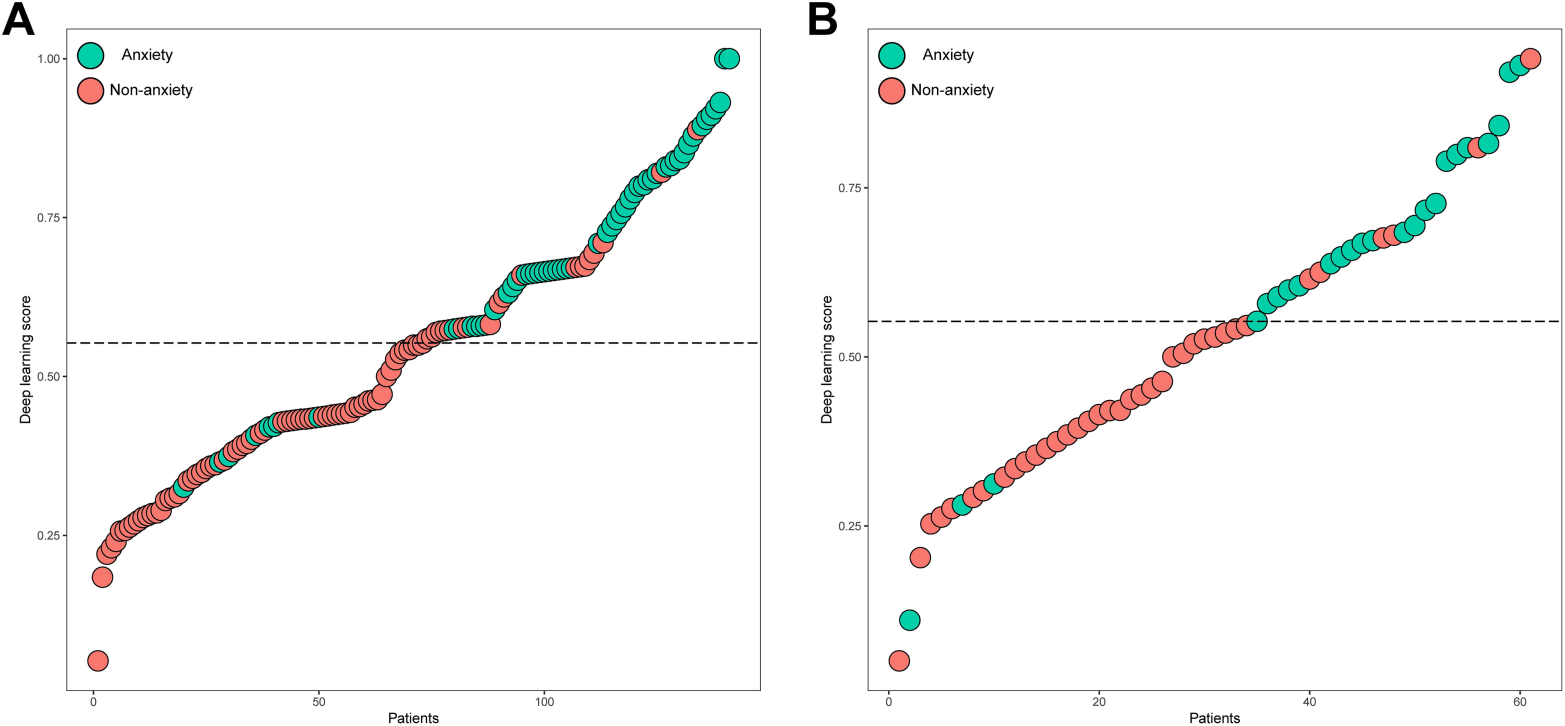
The distribution of the deep learning score and anxiety in the **(A)** training and **(B)** validation sets.

**Figure 4:**
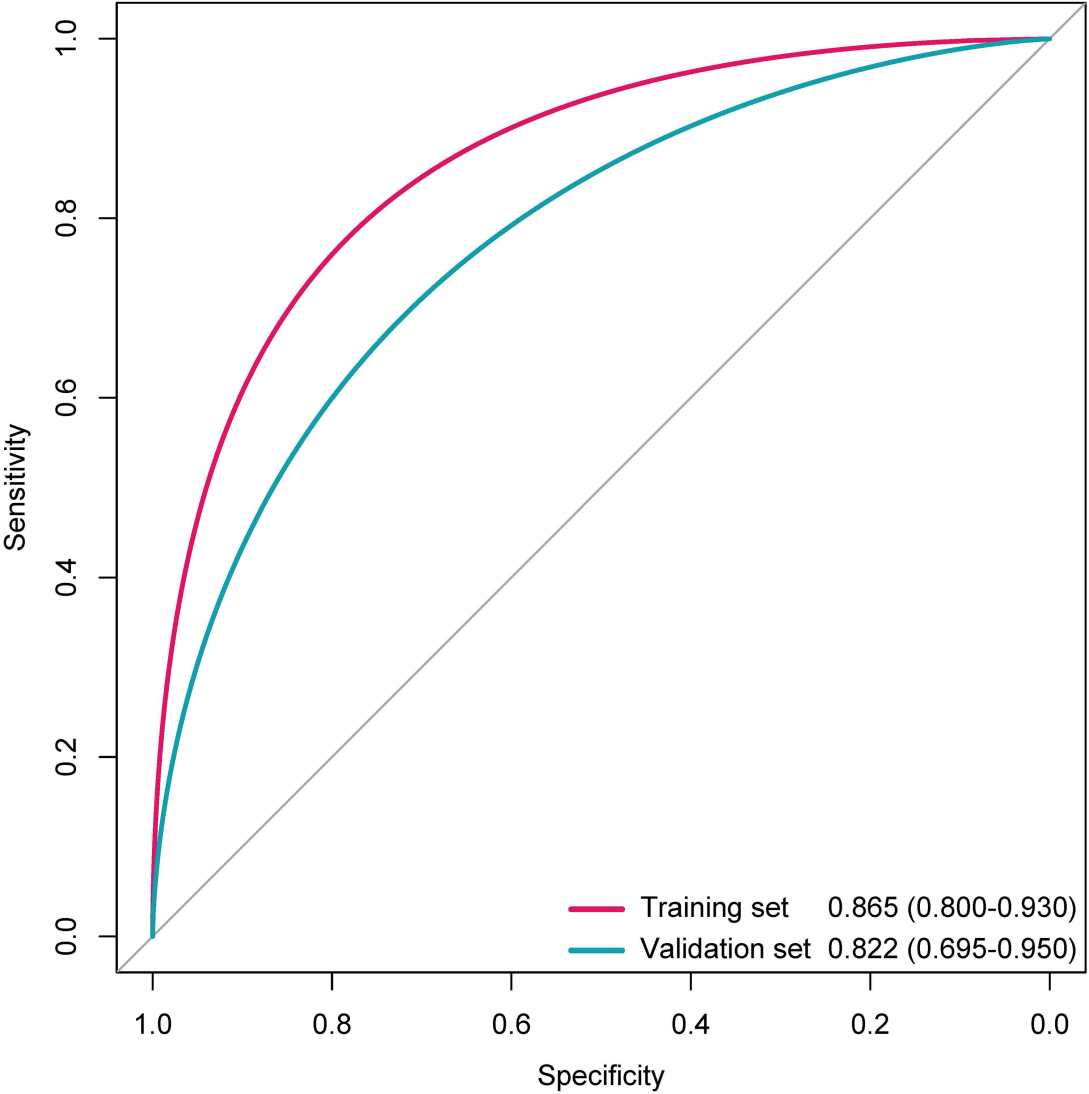
The ROC curves of the deep learning signature for predicting anxiety in the training and validation sets.

**Figure 5:**
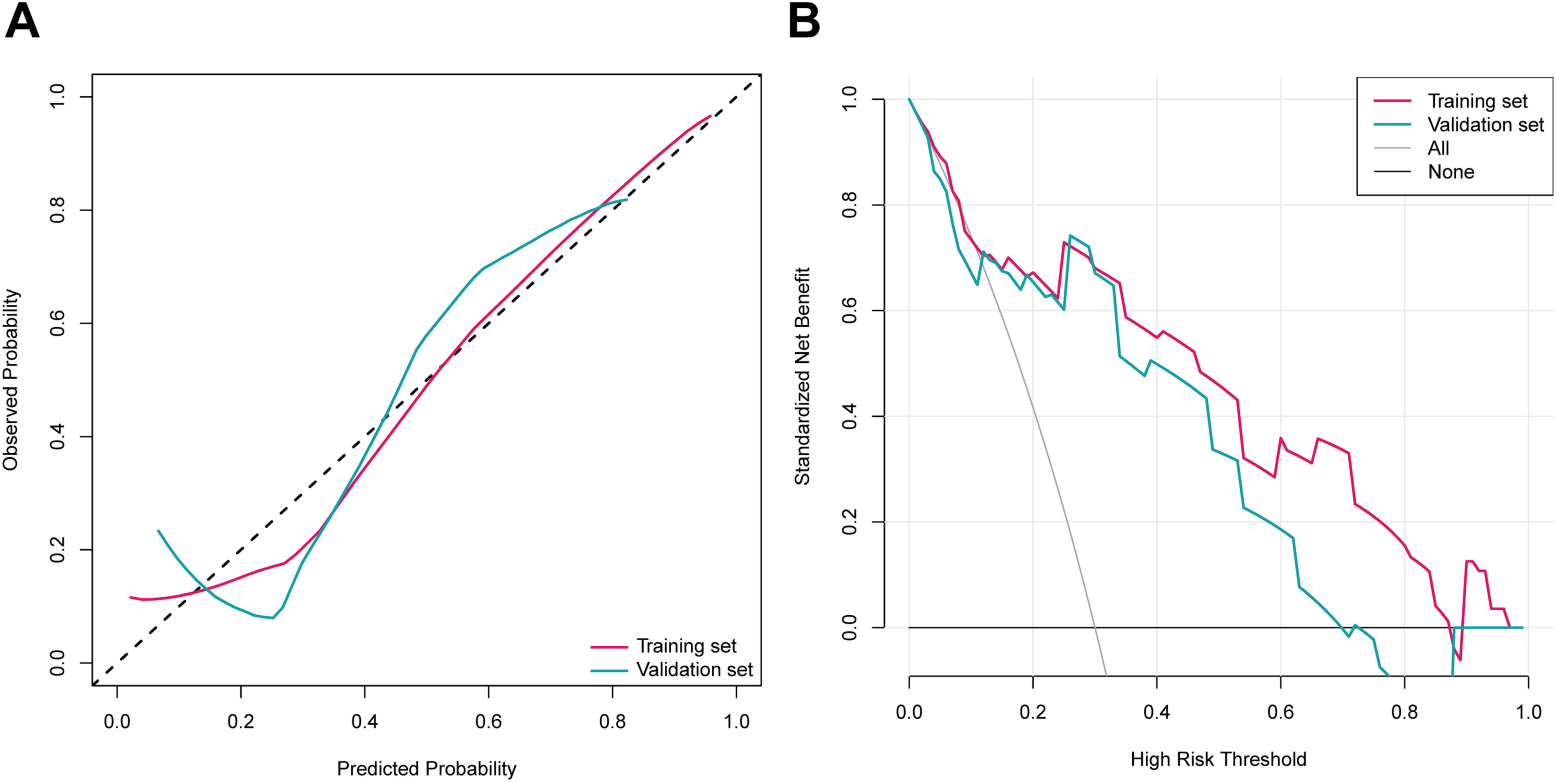
The **(A)** calibration curves and **(B)** decision curves of the deep learning signature.

The distributions of the deep learning score were illustrated in Figure 3, with the increase of deep learning scores, more patients experiencing postoperative anxiety were observed in the training and validation sets. As displayed in Figure 4, in the training set, the ability of the deep learning signature for predicting postoperative anxiety yielded an AUC of 0.865 (95% CI, 0.800–0.930). In addition, in the validation set, the deep learning signature achieved an AUC of 0.822 (95% CI, 0.695–0.950). The performance metrics were detailed in Table 3, the sensitivity, specificity, PPV, NPV, and accuracy of the deep learning signature was 82.1%, 85.6%, 79.3%, 88.0%, and 84.4% in the training set, and 83.3%, 83.8%, 76.9%, 88.6%, and 83.6% in the validation set. Moreover, as shown in Figure 5, the calibration curve and decision curve analyses indicated that the developed deep learning signature yielded satisfactory clinical usefulness in both training and validation set.

**Table 3:** Performance metrics of the deep learning signature.

Data set	Sensitivity	Specificity	PPV	NPV	Accuracy
Training set	82.1%	85.6%	79.3%	88.0%	84.4%
Validation set	83.3%	83.8%	76.9%	88.6%	83.6%

PPV, positive predictive value; NPV, negative predictive value.

## Discussion

Research indicates that anxiety is more common among lung cancer patients compared to healthy individuals (8, 11, 19). This trend persists even after surgery, with postoperative lung cancer patients showing higher anxiety levels than their healthy counterparts (20). In this investigation, the HADS-A scale was employed to assess anxiety in postoperative lung cancer patients. Additionally, a deep learning model was developed using brain MRI data, demonstrating that patients with higher deep learning scores exhibited elevated anxiety rates based on HADS-A. The model achieved an AUC of 0.822 in the validation set for predicting postoperative anxiety.

Given the significant proportion of anxiety in patients suffered from lung cancer, identifying risk factors is crucial for improving clinical management (9, 11). Prior studies have suggested that younger, non-surgical lung cancer patients are more prone to anxiety (11), while others have linked anxiety to dyspnea, severe pain, and diabetes (21). However, these studies often lacked comprehensive parameters, such as surgical details and tumor stage. To address this gap, our study incorporated a broader range of variables and found that tumor size and lymph node metastasis were independently associated with increased anxiety risk in postoperative patients. This may be attributed to pain and discomfort from advanced disease.

Anxiety adversely affects cancer prognosis, with studies linking it to higher mortality in breast and gastric cancer patients (22). Similarly, lung cancer patients with anxiety experience reduced survival rates (8, 12). Early identification of postoperative anxiety is therefore vital for optimizing treatment outcomes. Radiomics, which extracts detailed features from medical images, offers a promising tool for tumor diagnosis, prognosis, and treatment planning (13–16). However, this study has limitations. First, its single-center design limits the generalizability of the deep learning model, as external validation is lacking. Second, the dataset may introduce selection bias and confounding factors. Although multivariable regression was used to adjust for predictors, some variables' impacts remain unaddressed. Future studies with larger, more diverse cohorts are needed to confirm these findings and enhance the model's robustness.

## Conclusion

Our deep learning signature harbor the potential to serve as an effective biomarker to predict postoperative anxiety in patients receiving lung cancer surgery.

## Data Availability

The original contributions presented in the study are included in the article/Supplementary Material, further inquiries can be directed to the corresponding authors.

## References

[1] Nasim F Sabath BF Eapen GA. Lung cancer. Med Clin North Am. (2019) 103(3):463–73. 10.1016/j.mcna.2018.12.006 30955514

[2] Siegel RL Miller KD Jemal A. Cancer statistics, 2020. CA Cancer J Clin. (2020) 70(1):7–30. 10.3322/caac.21590 31912902

[3] Zhang Z Yang S Ma Y Zhou H Wu X Han J Consistency of recommendations for the diagnosis and treatment of non-small cell lung cancer: a systematic review. Transl Lung Cancer Res. (2021) 10(6):2715–32. 10.21037/tlcr-21-423 34295672 PMC8264323

[4] Bassanelli M Ramella S Zeuli M Ceribelli A. Radiotherapy and immunotherapy: the power of the teamwork for the treatment of NSCLC. Anticancer Res. (2022) 42(5):2241–7. 10.21873/anticanres.15704 35489718

[5] Yang Q Luo LC Li FM Yi Q Luo W. Survival outcomes of radiofrequency ablation compared with surgery in patients with early-stage primary non-small-cell lung cancer: a meta-analysis. Respir Investig. (2022) 60(3):337–44. 10.1016/j.resinv.2022.01.002 35172951

[6] Choi SH Yoo SS Lee SY Park JY. Anti-angiogenesis revisited: reshaping the treatment landscape of advanced non-small cell lung cancer. Arch Pharm Res. (2022) 45(4):263–79. 10.1007/s12272-022-01382-6 35449345

[7] Siwik CJ Phillips K Zimmaro L Salmon P Sephton SE. Depressive symptoms among patients with lung cancer: elucidating the roles of shame, guilt, and self-compassion. J Health Psychol. (2022) 27(5):1039–47. 10.1177/1359105320988331 33478252

[8] Arrieta O Angulo LP Núñez-Valencia C Dorantes-Gallareta Y Macedo EO Martínez-López D Association of depression and anxiety on quality of life, treatment adherence, and prognosis in patients with advanced non-small cell lung cancer. Ann Surg Oncol. (2013) 20(6):1941–8. 10.1245/s10434-012-2793-5 23263699

[9] Jung JY Lee JM Kim MS Shim YM Zo JI Yun YH. Comparison of fatigue, depression, and anxiety as factors affecting posttreatment health-related quality of life in lung cancer survivors. Psychooncology. (2018) 27(2):465–70. 10.1002/pon.4513 28755492

[10] Polański J Chabowski M Chudiak A Uchmanowicz B Janczak D Rosińczuk J Intensity of anxiety and depression in patients with lung cancer in relation to quality of life. Adv Exp Med Biol. (2018) 1023:29–36. 10.1007/5584_2017_50 28573442

[11] Yan X Chen X Li M Zhang P. Prevalence and risk factors of anxiety and depression in Chinese patients with lung cancer: a cross-sectional study. Cancer Manag Res. (2019) 11:4347–56. 10.2147/CMAR.S202119 31190999 PMC6514253

[12] Vodermaier A Lucas S Linden W Olson R. Anxiety after diagnosis predicts lung cancer-specific and overall survival in patients with stage III non-small cell lung cancer: a population-based cohort study. J Pain Symptom Manage. (2017) 53(6):1057–65. 10.1016/j.jpainsymman.2016.12.338 28063862

[13] Zhong Y She Y Deng J Chen S Wang T Yang M Deep learning for prediction of N2 metastasis and survival for clinical stage I non-small cell lung cancer. Radiology. (2022) 302(1):200–11. 10.1148/radiol.2021210902 34698568

[14] Gu Y She Y Xie D Dai C Ren Y Fan Z A texture analysis-based prediction model for lymph node metastasis in stage IA lung adenocarcinoma. Ann Thorac Surg. (2018) 106(1):214–20. 10.1016/j.athoracsur.2018.02.026 29550204

[15] Cong M Feng H Ren JL Xu Q Cong L Hou Z Development of a predictive radiomics model for lymph node metastases in pre-surgical CT-based stage IA non-small cell lung cancer. Lung Cancer. (2020) 139:73–9. 10.1016/j.lungcan.2019.11.003 31743889

[16] Zhong Y Cai C Chen T Gui H Deng J Yang M PET/CT based cross-modal deep learning signature to predict occult nodal metastasis in lung cancer. Nat Commun. (2023) 14(1):7513. 10.1038/s41467-023-42811-4 37980411 PMC10657428

[17] Zigmond AS Snaith RP. The hospital anxiety and depression scale. Acta Psychiatr Scand. (1983) 67(6):361–70. 10.1111/j.1600-0447.1983.tb09716.x 6880820

[18] Zhu J Qi Y Wu J. Medical SAM 2: segment medical images as video via segment anything model 2. 2024. arXiv:2408.00874.

[19] Guo C Huang X. Hospital anxiety and depression scale exhibits good consistency but shorter assessment time than Zung self-rating anxiety/depression scale for evaluating anxiety/depression in non-small cell lung cancer. Medicine. (2021) 100(8):e24428. 10.1097/MD.0000000000024428 33663054 PMC7909105

[20] Huang X Zhang TZ Li GH Liu L Xu GQ. Prevalence and correlation of anxiety and depression on the prognosis of postoperative non-small-cell lung cancer patients in north China. Medicine. (2020) 99(11):e19087. 10.1097/MD.0000000000019087 32176035 PMC7440182

[21] Park S Kang CH Hwang Y Seong YW Lee HJ Park IK Risk factors for postoperative anxiety and depression after surgical treatment for lung cancer†. Eur J Cardiothorac Surg. (2016) 49(1):e16–21. 10.1093/ejcts/ezv336 26410631

[22] Liu P Wang Z. Postoperative anxiety and depression in surgical gastric cancer patients: their longitudinal change, risk factors, and correlation with survival. Medicine. (2022) 101(11):e28765. 10.1097/MD.0000000000028765 35356898 PMC10684124

